# Avoidance of nocebo effects by coincident naming of treatment benefits during the medical interview for informed consent—Evidence from dynamometry

**DOI:** 10.3389/fpsyg.2022.923044

**Published:** 2022-08-09

**Authors:** Nina Zech, Matthias Schrödinger, Ernil Hansen

**Affiliations:** ^1^Department of Anesthesiology, University Hospital Regensburg, Regensburg, Germany; ^2^Department of Internal Medicine, District Hospital Wörth an der Donau, Wörth, Germany

**Keywords:** nocebo effect, suggestion, dynamometry, arm muscle strength, medical interview, informed consent

## Abstract

**Introduction:**

In the context of giving risk information for obtaining informed consent, it is not easy to comply with the ethical principle of “primum nihil nocere.” Carelessness, ignorance of nocebo effects and a misunderstood striving for legal certainty can lead doctors to comprehensive and brutal risk information. It is known that talking about risks and side effects can even trigger those and result in distress and nonadherence to medication or therapy.

**Methods:**

Recently, we have reported on significant clinically relevant effects of verbal and non-verbal suggestions on maximal muscular arm strength in healthy volunteers and in patients at two time points before surgery. Maximal strength during arm abduction was measured by dynamometry of the deltoid muscle group. Suggestions from clinical everyday life were formulated as presumed negative and neutral versions.

**Results:**

Here, we report on the effects of two versions of risk information in 45 patients. After sole mentioning risks of a puncture for the placement of a pain catheter, the maximal arm muscle strength was significantly reduced to 83% of baseline several days (T1), and to 84% the evening before surgery (T2). Strength was not significantly decreased and close to baseline at T1 and T2 when risks and benefits of a pain catheter were combined in one sentence. The difference between both versions was significant. With persistent normal distribution of values, the effect was due to uniform reactions of many patients, not to strong reactions of a few. High suggestibility and increase of anxiety with approaching surgery were identified as influencing factors for the neutralizing effect of modified wording.

**Conclusion:**

We not only suggest an alternative formulation for risk information to avoid nocebo effects but present an objective method to quantify and compare effects of different wordings. Thereby, we provide evidence that concurrently given positive aspects can neutralize negative effects during medical interview.

## Introduction

Risk information to achieve informed consent has been identified as the major cause of nocebo effects (Colloca and Miller, 2011; Häuser et al., 2012; Wells and Kaptchuk, 2012; Cohen, 2014). Talking about and explaining side effects of a medication or any other medical intervention may elicit or intensify those very same symptoms. Besides conditioning, i.e., learning from one’s own prior bad experiences, negative expectations, inadvertently induced during disclosure of adverse treatment effects, are the main origin of nocebo responses. The latter rarely are “non-specific” as often attributed to placebo/nocebo effects, but typically reflect exactly those discussed undesirable adverse reactions (Kaptchuk et al., 2006; Amanzio et al., 2009; Rief et al., 2009). Some of the physiological bases are now well understood, such as the involvement of certain brain areas or biochemical mediators (Benedetti et al., 2006). Nocebo effects not only add to the burden of illness, but also result in psychological distress, medication or therapy nonadherence, extra treatment visits and therapy of side effects, most of the latter incurring considerable extra costs (Barsky et al., 2002). In addition, negative expectations, hopelessness, and depressive reactions that can be induced by risks disclosure, are strong predictors of an unfavorable outcome of disease and therapy (Székely et al., 2007; Laferton et al., 2013; Tilbury et al., 2018). Finally, postponement or even refusal of a necessary medical intervention resulting from an inadequate disclosure of risk information represent further detrimental side effects of medical briefing for informed consent.

Therefore, physicians find themselves in the dilemma of respecting the Hippocratic doctrine of primum nihil nocere, i.e., “not to harm,” and the clinical reality of nocebo, i.e., “I will harm” (Miller and Colloca, 2011). Accordingly, most publications on the subject end with a call for a change and for improvements in the practice of providing information in order to obtain informed consent (Colloca, 2017; Evers et al., 2018; Howick, 2020). Nevertheless, any proposal to reduce expectancy-induced side effects has to respect the ethical principle that there is not only the right for autonomy, i.e., decisions after adequate information, but also the right for non-maleficence (Wells and Kaptchuk, 2012; Cohen, 2017; Fortunato et al., 2017). Although numerous studies have shown that side effects are significantly reduced in patients when risk information was withheld, nondisclosure is not an acceptable option (Daniels and Sallie, 1981; Myers et al., 1987; Mondaini et al., 2007). The question is not whether to provide information, but rather how to adequately provide that information. Moreover, lying and whitewashing are not allowed either because of the claim for truthfulness. Appropriate strategies must be based on knowledge of the mechanisms of nocebo effects, on established communication strategies, and on clinical experience (Schedlowski et al., 2015). Often proposals to reduce expectancy-induced side effects are rather general and hard to implement and verify, such as “enhanced treatment information,” “optimization of patient-clinician communication and relationship,” “managing patient’s treatment expectations,” and “selection and tailoring treatment to patients at risk” (Manaï et al., 2019). Despite several proposals, it still remains an open question as to what and how doctors should communicate to contribute to evidence-based practice and informed patient choice while minimizing nocebo effects, strongly calling for research (Miller and Colloca, 2011). The more so because only rarely has the effectiveness of the proposed approaches been measured and verified (Barnes et al., 2019; Fernandez et al., 2019; Pan et al., 2019), as necessary for an evidence-based approach.

We have recently proposed a measurement technique to qualify and quantify suggestion effects, namely alterations in maximal arm muscle strength in abduction (Zech et al., 2019, 2020). Muscle strength is a clinically relevant parameter with regard to early mobilization, risk of falling and sufficient breathing. Furthermore, the observed impairment of muscular performance could reflect a general “weakening effect” of negative suggestions (Hansen and Zech, 2019). With this objective test system adopted from physiology we have tested various verbal and nonverbal signals, designated as “suggestions,” from everyday clinical practice, and found significant weakening, or neutral reactions to alternative formulations, respectively. Herein we report on results with patients using two versions of disclosure of risk information for obtaining informed consent.

## Materials and methods

### Design and participants

In an experimental trial on 50 patients, we tested the effect of two versions of risk disclosure on maximal arm muscle strength during abduction. The data exclusively reported here were collected during a study on the effects of suggestions in the clinical context published recently (Zech et al., 2020). The sequence of tested interventions was randomized. After approval by the local ethics committee (EC University of Regensburg, Nr. 13-101-0030) the study was conducted at the University Hospital Regensburg, Germany. Patients between 18 and 70 years of age were considered for enrolment if they were to undergo elective surgery under general anesthesia no closer than 3 days either at the Departments of General Surgery, Neurosurgery, Otorhinolaryngology or Cranio-Maxillofacial Surgery. Participants had to be native German speakers and without relevant general pain (i.e., a Numeric Rating Scale NRS <5), and without pain or impairment of the dominant shoulder, arm or hand. Another exclusion criterion was a pre-existing severe systemic disease (ASA ≥ 3, according to the ASA physical status classification system of the American Society of Anesthesiologist). 50 patients fulfilling the inclusion criteria were enrolled after written informed consent and without financial compensation. A detailed description of this study can be found in the previous manuscript (Zech et al., 2020).

### Measurement of maximal muscle strength

**Figure 1:**
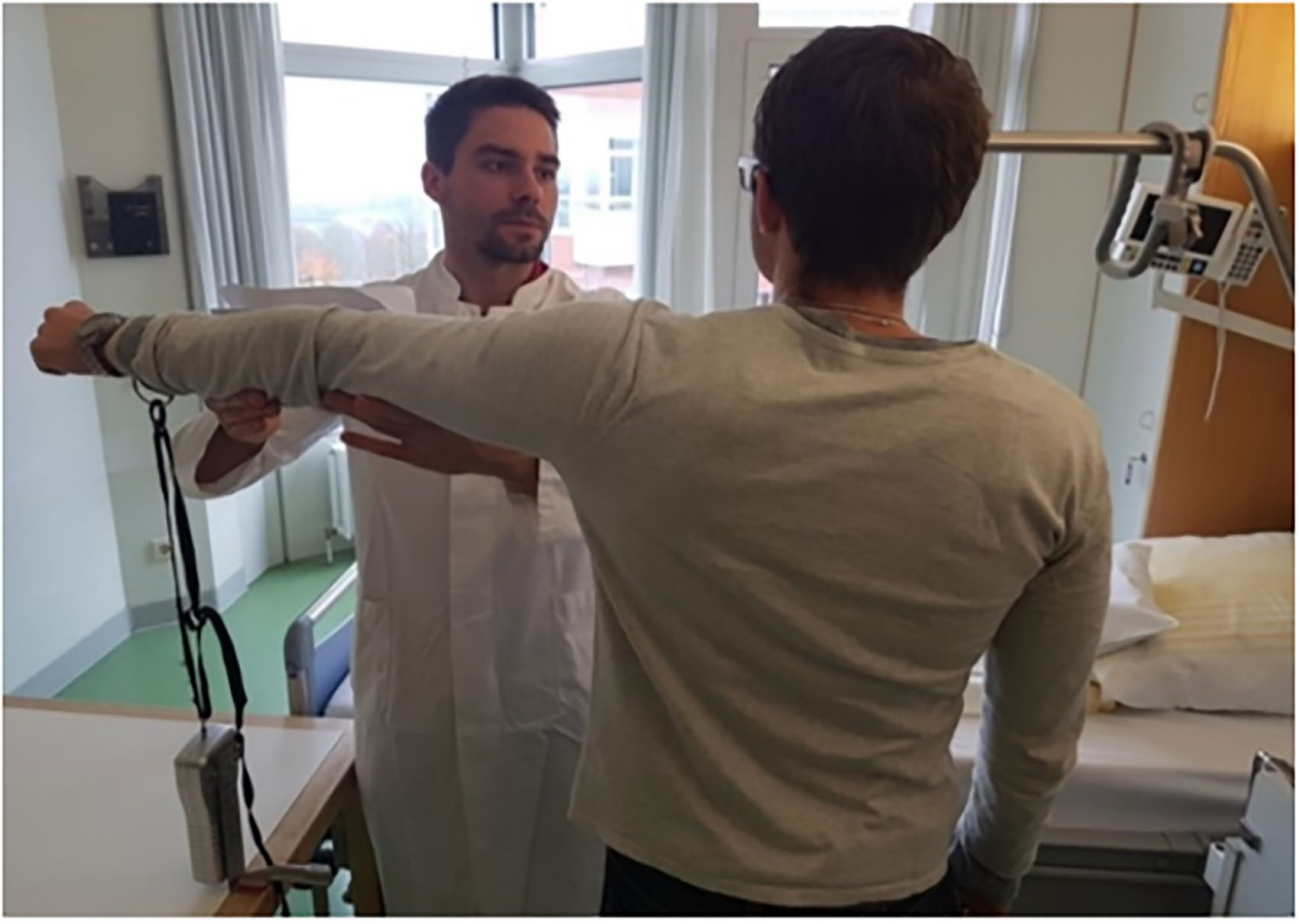
Test setup. For dynamometry of maximal arm muscle strength during abduction the patient stands upright, facing the tester, with the dominant arm stretched to the side and the wrist connected to the dynamometer by a band. Photo taken by NZ: MS with a patient, showing the standardized positioning.

Effects of suggestions on maximal muscle strength were measured at two timepoints: days before surgery (T1, minimum 3 days), and in the evening before surgery (T2). Maximal isometric contraction of the deltoid muscle group during arm abduction was tested by dynamometry, in a defined upright position with the dominant arm stretched out laterally (Figure 1). A dynamometer (FORCE GAUGE FM200, PCE Deutschland GmbH, Meschede, Germany) was used in the peak hold mode with a measurement accuracy of 0.5%.

Baseline was established for every patient by means of six initial measurements without suggestion followed by three to five such baseline measurements interspersed between tests of suggestions, adding up to a total of 9–11. The standardized instruction for this baseline measurement is given in Table 1. With a variation of ±6.3% of baseline values (Zech et al., 2019) maximal muscle strength measured under these conditions is a rather robust physiological parameter. However, individuals show a high range of variation in muscle strength. Therefore, the results of responses to suggestions were expressed as relative values, i.e., in percentage of the baseline value of each participant. All patients were tested by the same examiner (MS). Each test session lasted about 40–60 min, which was found feasible even for patients.

**Table 1:** Wording of the standardized instructions and verbal suggestions.

Category	Instruction	Risk disclosure	
		Version A	Version B
Baseline	“Now pull upward with maximal power. Now, one-two-three.”		
Suggestion	”Again, stand upright, lift your arm. Close your eyes. You are a patient in a hospital. You are faced with the following sentences. Take your time and let it affect you, and then pull upwards as hard as you can.”	“If you wish, we can place a pain catheter, with the **risk of infection, allergic reaction, and damage to blood vessels or nerves**.”	“We have the option of a local pain therapy. Even though there is a **risk of infection, allergic reaction, or damage to blood vessels or nerves**, you will have to take fewer pills, are more mobile, feel and recover better, and perhaps can go home sooner.”

Instructions were given from tape, suggestions face-to-face by the tester.

### Test of suggestion effect

Eighteen verbal and non-verbal suggestions out of clinical context were tested in two previous studies on healthy volunteers (Zech et al., 2019) and on patients (Zech et al., 2020). Here, the results of the two phrases designed to gain informed consent after risk information are reported. Patients listened to recorded instructions explaining the placement and functionality of the muscle test, whereas suggestions were given verbally, face to face. The wording of the instruction prior to suggestions, as well as the suggestions for risk information can be seen in Table 1. Version A was taken directly from everyday clinical practice and presumed to be negative and causing a nocebo effect. The alternative version B was formulated, considered to be positive and to elicit a neutral or placebo effect. After six baseline measurements, all suggestions were tested in a randomized order, using the software Randlist (Datinf GmbH, Tübingen), alternating a presumed negative version with a presumed neutral or positive version, to avoid cumulation effects. Tests were separated by breaks, arithmetical tasks and repeated determinations of blank values. In order to prevent incorrect measurements because of exhaustion an additional break was inserted, whenever a baseline value fell below 90% of the previous value, and the test repeated subsequently.

### Measurement of suggestibility and anxiety

Anxiety was measured with the state scale of the State–Trait-Anxiety-Inventory (STAI-S; Spielberger, 1985) with 20 test items in a German version (Laux et al., 1981) prior to the beginning of dynamometry. Evaluation took place at the two time points to draw conclusions about changes in anxiety over time with approaching operation date. With a range of 20 (“no fear”) to 80 (“worst fear”) points, the test evaluates the current situational anxiety. Anxiety is usually considered clinically relevant at a score >40, and at >55 rated relevant for psychiatric disorders (Knight et al., 1983; Addolorato et al., 1999). The difference between the scores at T2 and T1 is referred to as ΔSTAI-S and describes the change of anxiety between the two times of testing.

Suggestibility was evaluated with a 5-items short version of the Harvard Group Scale of Hypnotic Susceptibility (HGSHS-5:G; Riegel et al., 2021). The HGSHS has been established as an objective test method by Shor to determine the suggestibility of a single person or groups (Shor and Orne, 1963; Bongartz, 1985; Peter et al., 2015). The short version takes about 20 min instead of 60 min for the full version. Patients performed the test and the self-evaluation according to an audio file a few days after their operation. Based on the HGSHS-5:G score, patients were rated “low suggestible” with a score of 0 or 1, “medium suggestible” with a score of 2 or 3, and “high suggestible” with scores of 4 or 5.

### Statistical analyses

For statistical analyses IBM SPSS Statistics, version 26 was used. Normal distribution was tested according to Kolmogorow–Smirnow. Data are presented as mean ± standard deviation (SD) or as median (interquartile range) depending on the underlying distribution. A one-sample t-test was used to evaluate significant changes of relative maximal arm muscle strength (%) at different time points compared to the initial 100% (baseline value). A histogram using steps of 5% was used to present the distribution of the values. Repeated measure ANOVA was performed with relative maximal arm muscle strength as dependent variable and instructions (A vs. B) and time (T1 vs. T2) as within subject factors. Partial eta-squared was used as an estimate of the effect size. Univariate linear regression analysis was performed to test for the influence of age, anxiety, increase in anxiety and suggestibility score. A *p* level of <0.05 was considered to be statistically significant.

## Results

### Baseline characteristics

Missing data (only tested at T1 because patient declined, surgery rescheduled or canceled) resulted in the exclusion of five out of 50 recruited patients. Patient characteristics and baseline scores are presented in Table 2. The median time from T1 to day of surgery was 3 days, with a range of 3–25 days. 53% of the values were at day 3, the minimal allowed interval. The rest was distributed around day 6 before surgery. For two patients the interval was 25 days, specific for the type of surgery. Due to the individual physical condition of the patients, baseline muscle strength ranged from 18.8 N to 143.7 N. The reproducibility of the baseline values of each individual patient was high (variance ≤4.8%; 4.8% at T1 and 4.7% at T2, respectively). Baseline values did not differ significantly at T1 and T2 (*p* = 0.87). 23 patients showed a clinically relevant baseline state score (>40) for anxiety (STAI-S at T1), the score of five patients lay above the threshold (>55) relevant for psychiatric disorders. State anxiety raised significantly from days before surgery (T1) to the evening before the operation (T2) by 6.2 ± 8.9 (*p* < 0.001). Further analyses of determinants for anxiety and increase in anxiety can be found in Zech et al. (2020). Corresponding to the suggestibility score 12 patients (27%) were rated low suggestible and 10 patients (22%) high suggestible.

**Table 2:** Baseline characteristics of study population (*N* = 45).

Age (years)	Mean ± SD	43.8 ± 15.0
Female sex	*N* (%)	25 (56%)
State anxiety (STAI-S)		
Days before surgery (T1)	Mean ± SD	41.7 ± 10.3
Evening before surgery (T2)	Mean ± SD	47.9 ± 12.7
Suggestibility (HGSHS-5:G)	Median (IQR)	3 (1–3)
Days from first test (T1) to surgery	Median (IQR)	3.0 (3.0–7.0)
Baseline muscle strength (Newton)		
Days before surgery (T1)	Mean ± SD	65.0 ± 23.4
Evening before surgery (T2)	Mean ± SD	64.8 ± 23.5

STAI-S, State–Trait-Anxiety-Inventory (Spielberger, 1985).

HGSHS-5:G, 5-item Harvard Group Scale of Hypnotic Susceptibility (Riegel et al., 2021).

### Changes in maximal arm muscle strength after suggestion

**Figure 2:**
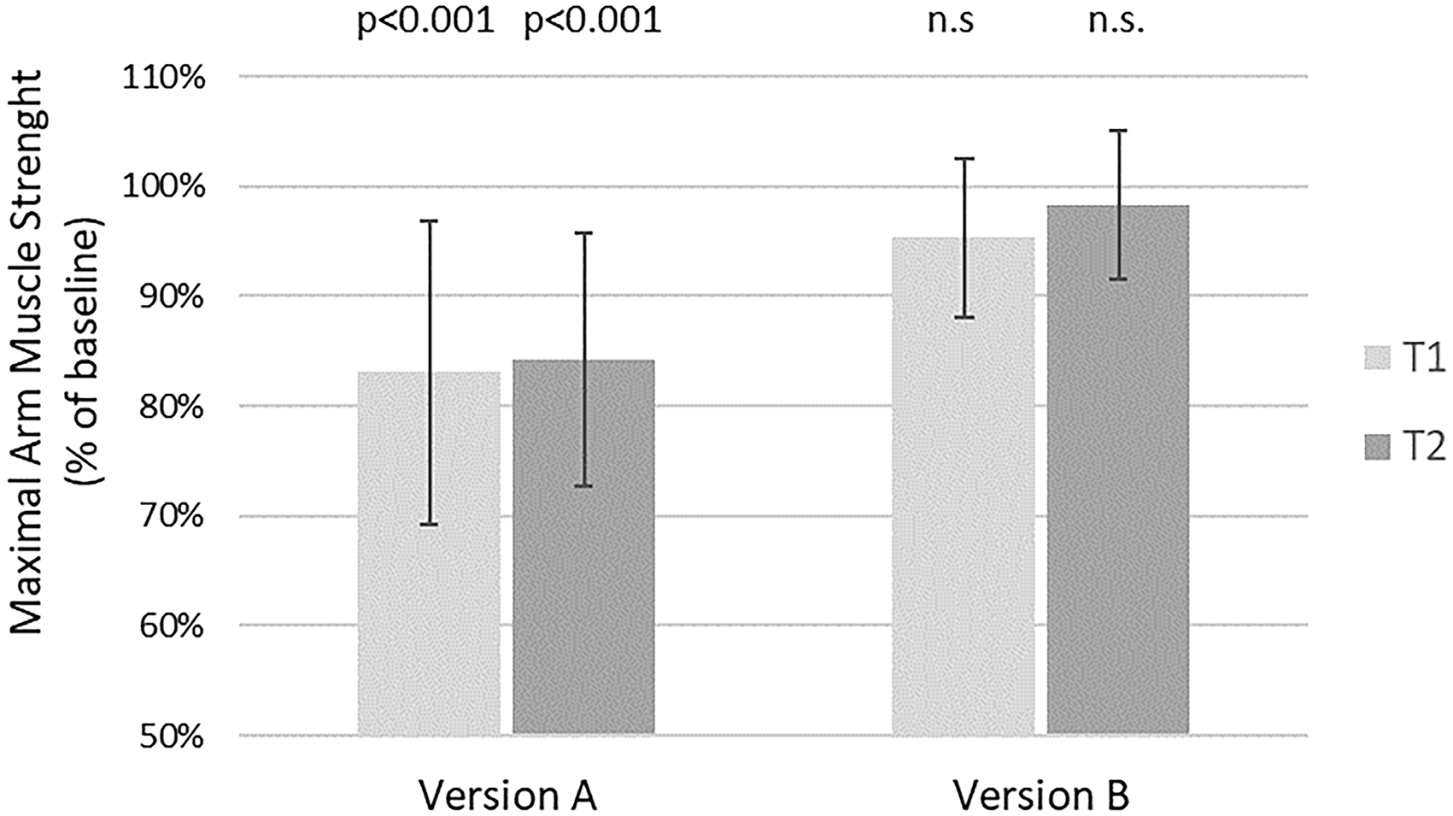
Effects of two different versions of risk disclosure on maximal arm muscle strength. After baseline dynamometry of arm abduction verbal suggestions were presented and measurement repeated. Version A: “If you wish, we can place a pain catheter, with the **risk of infection, allergic reaction, and damage to blood vessels or nerves**.” Version B: “We have the option of a local pain therapy. Even though there is a **risk of infection, allergic reaction, or damage to blood vessels or nerves**, you will have to take fewer pills, are more mobile, feel and recover better, and perhaps can go home sooner.” 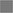
T1 = days before surgery 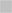
T2 = evening before surgery. Mean and SD of maximal arm muscle strength compared to baseline are given. P according to one sample *T*-test.

Version A to gain informed consent after risk information, taken from every day clinical practice and suspected to be negative, resulted in a highly significant reduction of maximal arm muscle strength at both time points, namely by 16.9% at T1 and by 15.7% at T2 compared to baseline, respectively (*p* < 0.001). There was no significant decline in muscle strength after version B at both time points. The reactions to version A did not differ significantly between time point T1 and T2, neither did the reactions to version B (Figure 2).

The difference between version A and version B was significant at T1 and at T2. ANOVA showed a significant effect of the wording of risk information but not of the time, nor of the interaction of the two (Table 3).

**Table 3:** Effect of wording and timing of risk information to obtain informed consent on maximal arm muscle strength.

	Relative maximal arm muscle strength (%)		
Time point	T1	T2	T2–T1
Version A	83.1 ± 14.1	84.3 ± 11.5	1.1 ± 13.0
Version B	95.6 ± 7.0	98.3 ± 6.8	2.7 ± 7.7
B–A	12.3 ± 13.2	14.0 ± 11.4	
*p*-values of repeated measures ANOVA: Difference between time points: *p* = 0.172, ηp2 = 0.04 Difference between versions: *p* < 0.001, ηp2 = 0.60 Interaction between time points and version: *p* = 0.380, ηp2 = 0.18			

After baseline measurements verbal suggestions were presented and dynamometry of arm abduction repeated. Mean and SD of relative values (compared to baseline) are given. Significance was tested by repeated measures ANOVA. T1 = days before surgery, T2 = evening before surgery; ηp2 = effect size measured by partial eta-squared.

### Distribution of values of maximal arm muscle strength at T2

**Figure 3:**
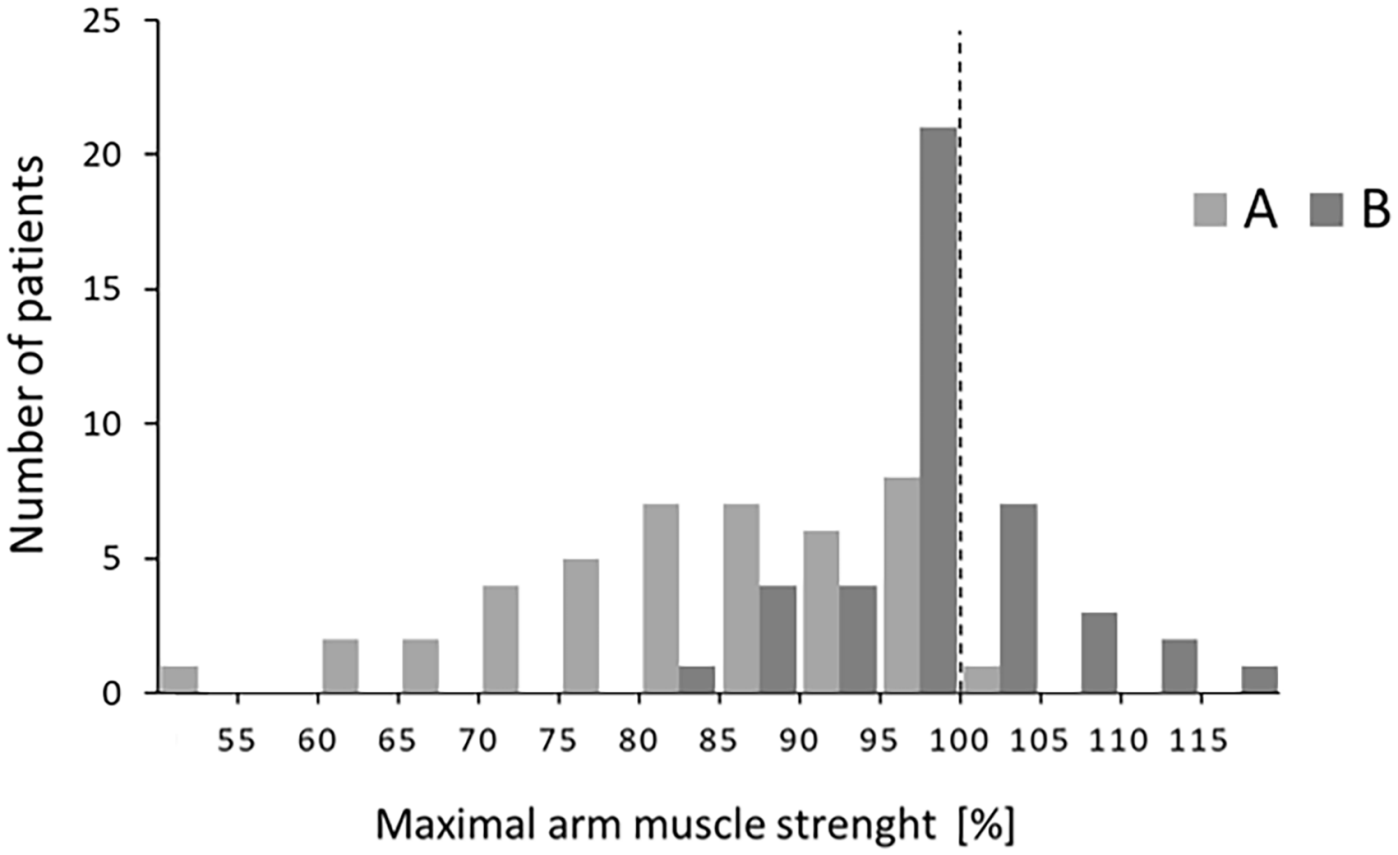
Distributions of relative muscle strength at T2 after the two versions A and B. Patients within a range of 5% points were grouped (e.g., 80%–84%).

To distinguish between the reaction of a few vs. that of most patients, a distribution of values is presented in Figure 3. Effects of both versions of risk information showed normal distribution. Relative muscle strength after version A ranged from 60% to 100%. Reactions to version B showed a narrower distribution from 80% to 115%. Six patients reached values higher than baseline.

### Influencing factors

**Figure 4:**
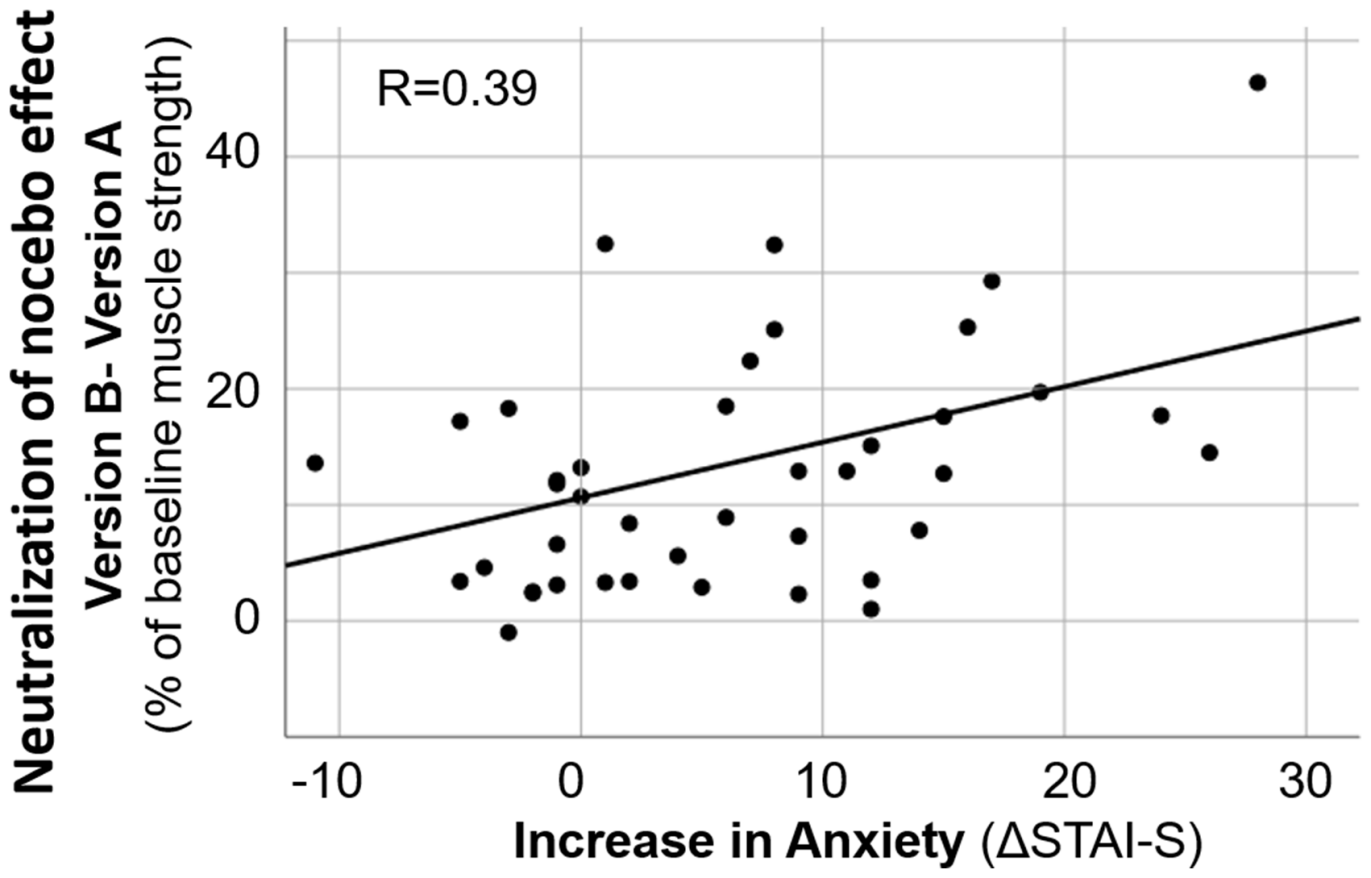
Linear regression analysis on the increase in anxiety and the decrease in negative effect of risk information on muscle strength by alternative formulation. ΔSTAI-S = STAI-S at T2 minus STAI-S at T1.

The influence of various factors (age, anxiety level, change in anxiety, and suggestibility score) on the effects of two versions of risk information on maximal muscle strength at the day before surgery (T2) was tested by linear regression analyses various factors. Both, the impact on the negative effect of the risk information (baseline – version A) and the impact on the neutralization of this negative influence by version B (muscle strength after version B – results after version A) were evaluated. Linear regression analyses showed that age, suggestibility score and anxiety score, as well the change in anxiety (ΔSTAI-S) did not significantly influence response of muscle strength to the suggestion. However, suggestibility score and ΔSTAI-S, i.e., the change in anxiety with approaching operation date, had a small but significant impact on the difference in the effects of the versions of risk information (Table 4). In patients with higher ΔSTAI-S maximal arm muscle strength showed improvement by version B compared to version A of risk information (Figure 4).

**Table 4:** Factors influencing the effect of risk information on maximal arm muscle strength and on its modification with an alternative formulation.

	Correlation coefficient *R* (*p*)	
	Version A	Version B–Version A
Age	−0.16 (0.299)	−0.19 (0.234)
HGHS-5 score	0.24 (0.124)	0.35 (0.023)
STAI-S	−0.07 (0.646)	0.07 (0.637)
ΔSTAI-S	0.28 (0.071)	0.39 (0.012)

Correlation coefficients are given according to linear regression analyses. STAI-S, State Anxiety Inventory at T2, ΔSTAI-S = STAI-S at T2 minus STAI-S at T1. Suggestibility: measured with the 5-items short version of the Harvard Group Scale of Hypnotic Susceptibility (HGSHS-5:G).

## Discussion

In the test setting of the present study, a usual wording of risk information resulted in reduction in maximal arm muscle strength. Thus, a negative effect on patients was objectively demonstrated and quantified. The weakening effect is confirmed by the measurement at two different time points, particularly days and at the evening before an operation.

### How to measure nocebo effects

Although the optimal demonstration of negative consequences of informed consent is an increase in the side effects discussed, such evidence needs high numbers of patients and a long observation period. Furthermore, most of the risks discussed in such medical informative interviews have multiple and complex origins and influencing factors, and the longer the time to their occurrence the more contributing factors join in. Parameters relevant for surgery for instance such as postoperative pain or nausea are dependent on the type of surgery, patient’s medication, type and course of anesthesia, and preposition and precondition of the patient. Besides the side effects addressed directly during the interview, nocebo effects of informed consent may also include more general medical complaints and burdens such as increased anxiety, hopelessness, hemodynamic instability, delay of wound healing, impaired immune response, and many others. In addition, different specific side effects allow no comparison, pain and nausea as nocebo effects cannot be contrasted quantitatively. Comparison of studies by effect size is nearly impossible, since different outcome parameters are measured: effects on symptom severity and duration, number of patients affected, number of side effects, different symptom qualities (e.g., various forms of pain). This heterogeneity in primary outcomes and their effect sizes hampers comparison of the effectiveness of different approaches for nocebo effect reduction.

In contrast, with the parameter maximal arm muscle strength nocebo effects can be qualitatively identified as such and can be objectively measured and quantified. An objective physiological measure is used instead of subjective psychological parameters such as pain score. Intensity of nocebo effects are studied instead of merely incidence. Moreover, with the use of one uniform parameter different nocebo effects can be compared. This allows also to study combinations of nocebo effects as they typically occur and sum up in clinical practice. Verbal and non-verbal signals interplay and are communicated all along during a hospital stay from admission to examination, from interview to risk assessment and information, from treatment to recovery. Comparison can also be made between different versions of a suggestion, like an alternative formulation of risk disclosure for informed consent in the present study. This allows different alternatives to be evaluated and thus communication be improved and optimized (Hansen and Zech, 2019). In addition, this parameter used in the present study and proposed for further nocebo research represents a physiological function of clinical relevance. Any impairment of muscle function is undesired, as enhancing the risk of falling, delay of mobilization after surgery, and insufficient respiration. The latter was confirmed by demonstration of respiratory muscle strength reduction after suggestions from clinical practice, including the risk information tested in the present study (Zech et al., 2022). Finally, the effects on maximal arm muscle strength were observed without verbal formulations directed to muscular function. While usually nocebo effects are tested within their specificity, e.g., pain after using the word “pain” or “stitch,” muscular function that was affected was not addressed in the tested risk information. Therefore, it was a more general effect that was observed, and even may be interpreted as marker for a “weakening” of the patient (Zech et al., 2019, 2020).

### How to detraumatize informed consent

According to the growing knowledge about nocebo effects that originate in the presentation of risk information to obtain informed consent, numerous proposals have been put forward to reduce or avoid the resulting negative consequences (Colloca, 2017; Klinger et al., 2017; Evers et al., 2018; Manaï et al., 2019; Howick, 2020; Zech et al., 2022). They reach from the idea of withholding the information about side effects (Daniels and Sallie, 1981; Myers et al., 1987; Mondaini et al., 2007), the mere talking about the existence of nocebo effects (Pan et al., 2019), or positive framing of information or side effects (Barnes et al., 2019), e.g., the occurrence of side effects as sign that the medication is active (Fernandez et al., 2019). However, rarely have the efficacy of such suggested approaches been tested. Reasons for this includes lack of standardizability of some of the proposed attempts, or the high number of patients necessary to evaluate rare side effects, that hinders scientific evaluation. Some publications are difficult to classify because the interventions are hardly described. For instance, for a “contextualized informed consent” urging for consideration of the specific patient, diagnosis and side effects, it is suggested to tailor information to the susceptibility of the patient and the degree of severity of the diagnosis, and to distinguish between unspecific and specific side effects (Wells and Kaptchuk, 2012). This approach has been challenged and designated unethical for containing partial withholding of information, and ineffective due to patients potentially gaining the information from other sources (Bromwich, 2012). This highlights the narrowness of the allowed corridor for framing: even if the treatment was authorized, the consent is considered invalid because the doctor exercised illegitimate control over the patient’s treatment decision by manipulating the given information.

In a meta-analysis of studies that have tested effectiveness of framing strategies, Barnes et al. reported positive effects in five of six studies with a low effective size of 0.09–0.24 (Barnes et al., 2019). Attribute framing, where side effects are expressed as “will not occur” (positive framing) or “will occur” (negative framing), had varying influence on number of patients affected, or number of side effects. However, the success was sometimes only short-lasting and only one of the studies involved patients. For example, following informed consent for an influenza vaccination fewer side effects and less absence from work were observed after positive framing (O’Connor et al., 1996). Two studies tested message framing, where in the positive version side effects are expressed as indicating that the drug works (Wilhelm et al., 2018; Fernandez et al., 2019). In a cold pressure task, framing had minimal impact on expectancies and incidence of side effects (Devlin et al., 2019).

A systematic review on effects of brief psychological interventions on adverse reactions found the strongest and most consistent effect from omitting risk information, no reduction of side effects by de-emphasizing, and mixed results from distraction, priming, or alteration of branding perception (Webster and Rubin, 2019). Informing about the nocebo effect has been shown to be able to reduce nocebo side effects after the intervention for a short time (Pan et al., 2019). Other attempts have been tested in experimental studies, and yet have to be translated to the clinical practice of presenting risk information for informed consent for a short time.

### Combining negative and positive expectations

Our attempt to neutralize negative impacts of risk information to obtain informed consent by simultaneous naming therapy benefits represent the most effective demonstrated so far, with an effect size Cohen’s d of 0.9 and 1.2 at the two test times, respectively. A comparison of the distributions of values shows that the neutralization was not due to the response of a few but to a uniform reaction of most patients. Closest to our approach comes an experimental trial of Bartels et al., where a nocebo effect induced by negative conditioning of itch to a color lamp was reduced by counterconditioning with a color light connected to a positive verbal suggestions (“The color will indicate an electrode that decreases the itch”) (Bartels et al., 2017). In a study on symptoms after windfarm sounds and media reports positive expectations (possible therapeutic effects of infrasound exposure) were able to attenuate effects from negative expectations (TV footage about health effects of wind turbines ultrasound), both when raised before or after the negative expectations (Crichton et al., 2014). The peculiarity and novelty of the present study is not the combination of negative and positive suggestions, but their simultaneous application. Information on both the benefits of therapy and the risks is also given to the patient in everyday clinical practice. However, most often they are separated by time or the medical discipline. The surgeon that has explained to the patient the benefit of the surgical therapy often only later talks about the associated risks, or the anesthetist gives information on risks of anesthesia without relying on those therapeutic benefits. In this study the negative suggestions connected with talking about side effects are presented together with the positive suggestions of treatment success, even in the same sentence. Maybe for the counterbalance of negative and positive expectations, and the resulting nocebo and placebo effects, simultaneity is essential. The aim of the interview for obtaining informed consent is to enable the patient to weigh up benefits and risks for a well-founded decision. This is achieved best when the patient has a look on both aspects at the same time instead of receiving information about treatment and its benefits separate from risk disclosure.

As the benefits of the proposed treatment are not the only positive aspect that can balance negative impacts of informed consent, various options to neutralize nocebo effects are listed in Table 5. Besides the principle of simultaneously naming something positive with the negative risk, the prophylactic measures taken to reduce or avoid the side effect can also be explained. Moreover, the careful monitoring during the intervention can be addressed, which facilitates immediate recognition of a developing adverse reaction and thereafter often allows rapid countermeasures and offers good treatment options. Sometimes the possibility of active patient participation to prevent side effects can be mentioned. That in addition gives back motivation and control to the patient. Probably these positive suggestions generate positive expectations and thereby compete with the negative expectations and nocebo effects induced by the risk information (Hansen and Zech, 2019). A limited capacity to process expectations simultaneously could be the reason.

**Table 5:** Options for neutralization of nocebo effect induction during risk disclosure by simultaneous presentation of positive aspects.

Positive aspects	Principle	Example
Treatment Benefit		“The peridural anesthesia carries a very small risk of neurologic impairment, but reduces pain and prevents the much more common adverse reactions like pneumonia, thrombosis or drug incompatibilities.”
Prophylaxis	Prophylaxis to prevent development of side effects	“We will carefully disinfect the skin where we do the surgery to prevent wound infection.”
Monitoring	Early detection of developing side effect	”That ECG-monitoring would immediately tell us should your diseased heart develop some arrhythmia, so we can start immediately with appropriate treatment.”
Treatability	Early treatment of progressing side effects and therapy of occurred harm	“We have all medication available to cope with and stop a developing allergic reaction.”
Patients Contribution	Active prophylaxis and monitoring	“If you repeat the breathing exercise I showed you often enough, you can contribute to the prevention of pneumonia.”

### Contributing factors

Various factors may have an impact on the development of nocebo effects and possibly on their neutralization (Table 4). In the present study no significant influence of age or gender was observed. Anxiety *per se* was also not a determining factor. However, an increase in state anxiety score with approaching operation date as deduced from ΔSTAI-S as well as hypnotic susceptibility score had an impact. Interestingly, both an rise in anxiety and suggestibility, exerted their influence not on the weakening effect of an ordinary risk information (baseline – version A) but on the neutralizing effect of a modified formulation (version B–version A) accounting for 15% of variance. However, suggestibility is such a minor determinant that the principle of neutralizing the nocebo effect by simultaneous addressing of positive aspects is not limited to high suggestible persons but can be used for all patients.

### Confirmation and future research

Our finding confirms results from a preceding study on healthy volunteers where the same test system was applied, and the same suggestions tested (Zech et al., 2019). Effect sizes are compared in Table 6. It is noticeable that the weakening effect of risk information disclosure (version A) was much more pronounced in patients compared to volunteers: −16.9 (at T1) and −15.7% (at T2) vs. −11.0% (Zech et al., 2019). This draws attention to the fact that nocebo effects measured in experimental settings may underestimate the real effects in clinical situations. Most importantly, neutralization by concomitant positive aspects was also more effective in the real clinical situation (for comparison of effect sizes see Table 6).

**Table 6:** Effectiveness of an alternative formulation of risk information to prevent weakening in healthy volunteers and in patients.

Risk information	Volunteers (Zech et al., 2019)	Patients (present study)	
		T1	T2
Version A vs. B			
Effect size, Cohen’s d (95% CI)	0.4 (0.2–0.7)	0.9 (0.6–1.3)	1.2 (0.8–1.6)

T1 = days before surgery, T2 = evening before surgery. Cohen’s calculated from paired Students *T*-test.

Reduction in maximal arm muscle strength measured in dynamometry, an easily available and feasible test system, can be used as surrogate marker for nocebo effect induction. It has proven effective in this and previous studies as a useful method for nocebo research (Zech et al., 2019, 2020). The approach to measure and quantify nocebo effects by a uniform physiological function like arm muscle strength not only allows for comparison of negative influences but also of alternative wordings for a better communication. Thereby, various attempts to avoid nocebo effects can be tested as well as positive suggestions. Moreover, combinations of verbal interventions can be evaluated. Altogether, the approach used and proposed here allows improvement of doctor-patient communication and interviews for informed consent according to scientific and comprehensible principles (Crichton et al., 2014).

Nevertheless, the proposed alternative positive aspects to combine with the risk information such as prophylactic and therapeutic measures to prevent or treat side effects (as presented in Table 5) have yet to be verified in studies. So do their combinations. In general, it must be said that the many well-considered and promising proposals of improvements in preventing nocebo effects after interviews for informed consent found in the recent literature still have to be measured, quantified and to show their effectiveness in both experimental and clinical studies.

## Data availability statement

The raw data supporting the conclusions of this article will be made available by the authors, without undue reservation.

## Ethics statement

The studies involving human participants were reviewed and approved by EC University of Regensburg, Nr. 13-101-0030. The patients/participants provided their written informed consent to participate in this study. Written informed consent was obtained from the individual(s) for the publication of any identifiable images or data included in this article.

## Author contributions

NZ: study design, application for ethic committee approval, literature search, participant recruitment, data collection and analysis, and preparation of the manuscript. EH: study plan and design, supervision, literature search, data analysis, preparation of figures, tables, and manuscript, and correction of manuscript. MS: participant recruitment and data collection and analysis. All authors contributed to the article and approved the submitted version.

## Conflict of interest

The authors declare that the research was conducted in the absence of any commercial or financial relationships that could be construed as a potential conflict of interest.

## Publisher’s note

All claims expressed in this article are solely those of the authors and do not necessarily represent those of their affiliated organizations, or those of the publisher, the editors and the reviewers. Any product that may be evaluated in this article, or claim that may be made by its manufacturer, is not guaranteed or endorsed by the publisher.
